# Genetic basis of hyperlysinemia

**DOI:** 10.1186/1750-1172-8-57

**Published:** 2013-04-09

**Authors:** Sander M Houten, Heleen te Brinke, Simone Denis, Jos PN Ruiter, Alida C Knegt, Johannis BC de Klerk, Persephone Augoustides-Savvopoulou, Johannes Häberle, Matthias R Baumgartner, Turgay Coşkun, Johannes Zschocke, Jörn Oliver Sass, Bwee Tien Poll-The, Ronald JA Wanders, Marinus Duran

**Affiliations:** 1 Department of Clinical Chemistry, Laboratory Genetic Metabolic Diseases, Academic Medical Center, University of Amsterdam, Meibergdreef 9, Amsterdam, AZ 1105, The Netherlands; 2 Department of Pediatrics, Emma Children’s Hospital, Academic Medical Center, University of Amsterdam, Amsterdam, The Netherlands; 3 Department of Clinical Genetics, Academic Medical Center, University of Amsterdam, Amsterdam, The Netherlands; 4 Department of Pediatrics, Erasmus Medical Center, Rotterdam, The Netherlands; 5 University 1st Department of Pediatrics, Metabolic Laboratory, Hippocration General Hospital of Thessaloniki, Thessaloniki, Greece; 6 Division of Metabolism, University Children’s Hospital Zürich, Zürich, Switzerland; 7 Division of Clinical Chemistry and Biochemistry, University Children’s Hospital Zürich, Zürich, Switzerland; 8 Unit of Metabolism, Hacettepe University Faculty of Medicine, Ankara, Turkey; 9 Division of Human Genetics, Medical University Innsbruck, Innsbruck, Austria; 10 University Children’s Hospital Freiburg, Freiburg, Germany

**Keywords:** Inborn errors of metabolism, Hyperlysinemia, Lysine, Contiguous gene deletion syndrome

## Abstract

**Background:**

Hyperlysinemia is an autosomal recessive inborn error of L-lysine degradation. To date only one causal mutation in the *AASS* gene encoding α-aminoadipic semialdehyde synthase has been reported. We aimed to better define the genetic basis of hyperlysinemia.

**Methods:**

We collected the clinical, biochemical and molecular data in a cohort of 8 hyperlysinemia patients with distinct neurological features.

**Results:**

We found novel causal mutations in *AASS* in all affected individuals, including 4 missense mutations, 2 deletions and 1 duplication. In two patients originating from one family, the hyperlysinemia was caused by a contiguous gene deletion syndrome affecting *AASS* and *PTPRZ1*.

**Conclusions:**

Hyperlysinemia is caused by mutations in *AASS*. As hyperlysinemia is generally considered a benign metabolic variant, the more severe neurological disease course in two patients with a contiguous deletion syndrome may be explained by the additional loss of *PTPRZ1*. Our findings illustrate the importance of detailed biochemical and genetic studies in any hyperlysinemia patient.

## Background

**Figure 1:**
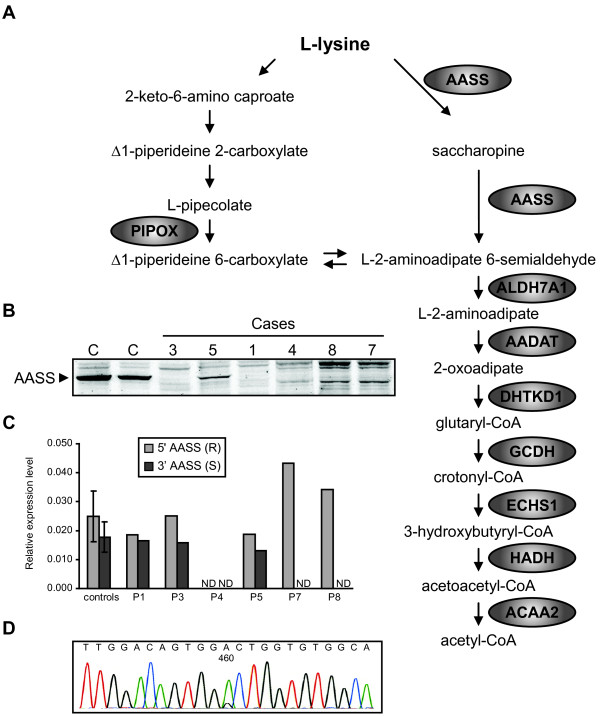
**Molecular and biochemical studies in hyperlysinemia patients. **(**A**) Schematic representation of the lysine degradation pathway. Lysine can be degraded via two pathways. The pathway with L-pipecolic acid as an intermediate operates in brain and starts with oxidative deamination. The main pathway in other organs, however, proceeds via deamination with saccharopine as an intermediate. All genes known to operate in this pathway are indicated. *ALDH7A1 *is deficient in children with pyridoxine-dependent seizures [3]. *GCDH* is deficient in glutaric aciduria type 1 [4]. The intermediate 2-oxoadipate is metabolized by 2-oxoadipate dehydrogenase, resembling the TCA cycle enzyme complex 2-oxoglutarate dehydrogenase. The E1 subunit of this complex is encoded by *DHTKD1* and is deficient in 2-aminoadipic and 2-oxoadipic aciduria [5]. (**B**) Immunoblot analysis of fibroblast homogenates of hyperlysinemia cases. Cell lysates of 2 control subjects and hyperlysinemia cases were resolved by SDS-PAGE (30 μg of protein) blotted onto nitrocellulose and analyzed with a polyclonal antibody against AASS. (**C**) Relative expression levels of AASS in fibroblasts determined using qPCR with a primer sets specific for the 5^′^ (R) and 3^′^ (S) part of the cDNA. Average and SD are provided for 3 control fibroblasts. ND denotes not detectable. Expression was normalized to the level of cyclophilin B (*PPIB*). (**D**) Electropherogram showing that the heterozygous c.460G>A mutation in patient 5 appears homozygous at the cDNA level, indicating nonsense mediated decay of the other allele (c.2076dup).

Hyperlysinemia is an autosomal recessive inborn error of metabolism caused by a defect in the major catabolic pathway of the essential amino acid L-lysine. This pathway is primarily active in the liver and leads to the production of acetyl-CoA (Figure 1A). In the first step, lysine and α-ketoglutarate are converted into saccharopine by lysine-ketoglutarate reductase (LKR, EC 1.5.1.8). Saccharopine is then oxidized to α-aminoadipic semialdehyde and glutamate by saccharopine dehydrogenase (SDH, EC 1.5.1.9). In animals, but also other eukaryotes, both enzyme activities are catalyzed by a single mitochondrial bifunctional enzyme named α-aminoadipic semialdehyde synthase [1], which is encoded by the gene *AASS*. One mutation in *AASS* has been reported to cause hyperlysinemia (c.1601_1609del; p.C534X [2]).

Hyperlysinemia is characterized by elevated plasma lysine levels that exceed 600 μmol/L and can reach up to 2000 μmol/L (adult reference range 111–248 μmol/L) [6-9]. Cases in which the plasma lysine levels are elevated, but remain below 600 μmol/L, are often caused by decreased availability of α-ketoglutarate [10], which occurs in urea cycle disorders, pyruvate carboxylase deficiency, methylmalonic aciduria and propionic aciduria. As a consequence of the accumulating lysine, several alternative biochemical reactions take place. Lysine can be used in place of ornithine in the urea cycle resulting in the production of homoarginine [11]. In addition, N-ε-acetyl-L-lysine, N-α-acetyl-L-lysine and pipecolic acid (Figure 1A) accumulate as a result of the use of alternative or detoxifying biochemical pathways [11,12]. Hyperlysinemic individuals may also have saccharopinuria, when the SDH is deficient in combination with a preserved LKR activity (also known as hyperlysinemia type 2 [13]).

Although hyperlysinemia was initially associated with neurological damage and mental retardation, later reports questioned this relationship. This was mainly based on a review of 10 individuals identified with hyperlysinemia in newborn screening programs (4 cases), family surveys of a previously diagnosed case (4 cases) and during the investigation of a boy and his family for short stature (2 cases). Cases were rejected if the hyperlysinemia was diagnosed during investigation of a patient presenting with a neurological or intellectual impairment. In none of the identified 10 cases adverse effects could be attributed to hyperlysinemia [14]. Furthermore, it is unclear whether dietary restriction of lysine is beneficial [11,15], and a child with no clinical manifestations of hyperlysinemia has been born to an affected mother [14]. These observations indicate that hyperlysinemia most likely represents a benign metabolic variant and illustrates that the association of nonspecific clinical signs and symptoms with biochemical abnormalities can be coincidental. Indeed it is currently unknown whether lysine or lysine-derived metabolites are toxic. In vitro, high concentrations of lysine (5 mM) induced oxidative damage to proteins and lipids, decreased oxidized glutathione and inhibited cytosolic creatine kinase activity [16,17]. Intrastriatal injection of lysine in rats did not affect creatine kinase, but inhibited synaptic Na^+^,K^+^-ATPase activity, induced lipid peroxidation and decreased glutathione level [18]. It is currently unclear how these findings can be related to clinical manifestations in patients with hyperlysinemia.

Despite this, biochemical diagnoses of hyperlysinemia are still made. Moreover, exome and whole genome sequencing projects are expected to identify variants in the *AASS* gene of unknown significance. To better define the genetic basis of hyperlysinemia, we collected clinical findings, and biochemical and molecular data in a cohort of 8 patients with distinct neurological features and hyperlysinemia.

## Methods

### Collection of the cohort and case reports

The hyperlysinemia patients described here (Table 1) were collected over the years and material was sent to a laboratory for metabolic screening and confirmatory testing. In most cases, patients displayed neurological symptoms for which a metabolic cause was suspected or had to be excluded. The studies described in this report have been performed as part of patient care and according to Dutch law do not need additional approval of a medical ethical committee if used for retrospective anonymous evaluation.

**Table 1:** Biochemical and molecular findings in 8 cases diagnosed with hyperlysinemia

**Case**	**Plasma lysine (μmol/L)**	**AASS immunoblot**	**Allele 1 (coding effect)**	**Allele 2 (coding effect)**
1	992 – 1688	Undetectable	c.194G>A (p.R65Q)	c.1256T>G (p.L419R)
2	787 – 1253	ND	c.194G>A (p.R65Q)	c.1256T>G (p.L419R)
3	1090 – 1326	Undetectable	c.194G>A (p.R65Q)	c.194G>A (p.R65Q)
4	868 – 1461	Undetectable	Deletion of exon 1 (no mRNA)	Deletion of exon 1 (no mRNA)
5	1554	Decreased	c.460G>A (p.A154T)	c.2076dup (p.P693SfsX10)
6	1054 – 1303	ND	c.2155A>G (p.T719A)	c.2155A>G (p.T719A)
7	2029	Undetectable	Deletion of exon 20-24	Deletion of exon 20-24
8	602	Undetectable	Deletion of exon 20-24	Deletion of exon 20-24

ND denotes not done.

Case 1 is the first child of non-consanguineous parents. He presented at age 6 months with psychomotor retardation and failure to thrive with vomiting due to severe gastro oesophageal reflux. In addition, there were craniosynostosis, microcephaly, a slightly dysmorphic face, and early development of spastic diplegia rapidly progressing to tetraparesis. Diagnosis of hyperlysinemia was established following the detection of elevated pipecolic acid. At age 4 years, motor development was severely retarded with inability to sit or stand. At this age, treatment with botulinum toxin was started and continued since then. Under this treatment, the patient learned to stand up and walk single steps with support. His expressive speech development is also largely retarded and he communicates with sign language. The EEG in this patient was normal as was the brain MR imaging at an age of 4 years. He is now 8 years old and severely affected by global retardation, spastic tetraparesis and microcephaly.

Case 2 is the younger brother of case 1 and showed a similar but less severe clinical course. At age 2 years, he walked on tiptoes as the first sign of a cerebral movement disorder. Treatment with botulinum toxin injections was started at the age of 4 years supporting the motor development. Currently at 7 years of age, the patient is able to walk and he can speak in full sentences. As in his older brother, the patient was affected by failure to thrive. Both brothers were treated with a lysine restricted diet, however, without any obvious benefit.

Case 3 is a boy of consanguineous parents (first degree cousins). He had an epileptic event during fever at the age of 6 months and was treated with anti-epileptic drugs for 2 months. From the age of 3 years developmental delay with spastic diplegia and behavioral disturbances became apparent. Magnetic resonance imaging (MRI) of the brain was normal. At the age of 4.5 years there was one single epileptic event without fever. A healthy sister did not have hyperlysinemia.

Case 4 is the third child of healthy consanguineous parents (first degree cousins). In two older healthy siblings hyperlysinemia was excluded. He came to clinical attention at the age of 10 months because of mild psychomotor retardation. Screening for urinary amino acids showed highly elevated lysine (7307 mmol/mol creatinine) in the absence of saccharopinuria. On physical examination, he displayed muscular hypotonia with brisk tendon reflexes. An EEG was normal. The patient was treated for three years with a strict low protein diet but this was relaxed and eventually stopped at age 5 years without obvious disadvantage to the neurological status. Currently at an age of 10 years, cognition is borderline normal with a debatable need for special education such as speech therapy and occupational and physiotherapy. The neurological examination including the muscle tone is normal.

Case 5 is the younger sister of a previously reported affected boy [11]. Both were diagnosed with epilepsy, intellectual disability and behavioral problems. The third child in this sibship was healthy and had normal lysine levels. The patient came to the attention of a child neurologist at the age of two years because of seizures. Her EEG was abnormal with epileptic changes. A CT at the age of 18 showed widened ventricles. Several seizures were observed during childhood which responded well to standard anti-epileptic medication (valproate, carbamazepine). Her intelligence was low normal (IQ 86), but she was referred to an institution because of behavioral problems.

The patient had recurrent pneumonias as a result of atelectasis of the left lung due to a congenital hypoplasia of the left pulmonary artery. The left lung was surgically removed at the age of five. The pulmonary problems never resolved completely; at the age of 19, following influenza vaccination, she again developed a severe pneumonia and as a consequence of this a chronic polyneuropathy became manifest. Her physical inabilities at this stage necessitated admission to a rehabilitation center. After this episode, she was lost for follow-up.

Case 6 is a boy diagnosed with intellectual and motor disability of unknown cause. His development was impaired in all areas; motor development, language, and behavior. He was hyperactive and restless, but with little regulation. Attention deficit disorder was not excluded. He had strabism, microcephaly, small stigmata, thin eyebrows, a flat philtrum and a flat nose, but no syndrome could be attributed. Prenatal exposure of the child to toxic compounds was suspected. The child has been under the care of the youth welfare service since birth and was placed with foster parents before he was ten months old. It was the intention to let the child attend a kindergarten with special support. Urine lysine values were 2386–3196 mmol/mol creatinine. Saccharopine was noted in urine (11.0 mmol/mol creatinine) and serum (3.5 μmol/L).

Case 7 is a boy of consanguineous parents (first degree relatives). The first child of the parents was a healthy male. The patient was born after an uneventful pregnancy and delivery at 38 weeks of gestation. Birth weight and length were normal, but head circumference was 34 cm (< 5 p). He had no spontaneous breathing after birth and was admitted to the neonatal intensive care unit with seizures, respiratory difficulties, hypotonia, and bradycardia at 2 days of life. Physical examination revealed a prominent hypotonia, mild dehydration, increased deep tendon reflexes, absence of sucking, Moro, and grasping reflexes. He had dysmorphic features such as microcephaly, an underdeveloped antihelix and helix, hypotelorism and a high arched palate. Cranial MRI showed a subacute haemorrhage in the 4th and lateral ventricles, a mild subdural haemorrhage in the occipital lobe and haemorrhages in sinus transversus and sagittalis superior. The patient was diagnosed with hyperlysinemia and started a special diet containing 50 mg/kg/day lysine including 2–25 g/kg/day protein and 140–150 kcal/kg/day energy and was given antiepileptic drugs including phenobarbital. He had feeding and swallowing dysfunction and was fed with a nasogastric tube. Severe spasticity and opisthotonic posturing developed in the follow up period. Unfortunately, the patient did not respond to treatment and no improvement in his signs and symptoms occurred and he died at 6 months of age.

Case 8 was born as the first child of consanguineous parents with a birth weight of 2800 g after an uneventful pregnancy at 38 weeks. His twin died at 3 days of life due to pulmonary hemorrhage. He was the cousin of case 7. Pneumonia and convulsions of generalized tonic type manifested on the second day of life. During the follow up in a local hospital for 2 months he was treated with phenobarbital. At 16 months of age, the patient was referred to the metabolic unit. Physical examination revealed microcephaly, an enlarged right ear, a high palate, psychomotor retardation, increased deep tendon reflexes, and decreased subcutaneous fat tissue. The patient did have head control but could not sit with or without support. A brain MRI was normal. His diet contained protein and calories appropriate for his age and weight. At 4.5 years the boy was spastic and could sit with support for a short time. He is 13 years old now and seizures were controlled with anticonvulsants.

### Patient cell lines

Primary skin fibroblasts from patients and controls were cultured in DMEM medium with 4.5 g/L glucose and L-glutamine (Lonza) supplemented with 10% fetal bovine serum (Lonza), 100 U/mL penicillin, 100 μg/mL streptomycin, fungizone (Life Technologies) and 25mM Hepes in a humidified atmosphere of 5% CO_2_ at 37°C.

### Enzyme activity measurements

Fibroblast pellets were resuspended in PBS and homogenized by sonication (twice on ice, 40J at 8W output). LKR was assayed spectrophotometrically at 340 nm by measuring the oxidation of NADPH. The reaction mixture contained 50 mM HEPES pH7.4, 10 mM α-ketoglutarate, 0.3 mM NADPH and 0.25% Triton X-100. The reaction was started with L-lysine (15 mM final concentration). SDH was assayed spectrophotometrically at 340 nm by measuring the formation of NADH. The reaction mixture contained 50 mM tricine pH8.5, 5 mM NAD and 0.1% Triton X-100. The reaction was initiated by adding saccharopine to a final concentration of 3.5 mM (stock solution of 35 mM in 100 mM tricine, pH 8.0). Both assays were performed kinetically on a COBAS FARA II centrifugal laboratory analyzer (Roche).

### *AASS* genome sequencing and cDNA analysis

All exons, plus flanking intronic sequences of the *AASS* gene (NM_005763), were sequenced after amplification by PCR from genomic DNA. All forward and reverse primers (Sets A to Q, Table 2) were tagged with a –21M13 or M13rev sequence, respectively. PCR fragments were sequenced in two directions using –21M13 and M13rev primers by means of BigDye Terminator Cycle Sequencing (v1.1, Applied Biosystems, Foster City, CA) and analyzed on an Applied Biosystems 3130*xl* or 3730*xl* DNA analyzer.

**Table 2:** Primer sets used for AASS mutation analysis

**Amplicon**	**Sequence (5**^**′**^**>3**^**′**^**)**^**1**^	**Exons**	**Regions of cDNA**
A	[-21M13]-cgattggcagatgagaaggt	1	(c.–221-c.–16)
	[M13-Rev]-atctccaccgcatctcacag		
B	[-21M13]-cacttgacatcccagttttcc	2	(c.–15-c.210)
	[M13-Rev]-ttcctcagctggagtaagca		
C	[-21M13]-tgttgtgcctttgctacaca	3	(c.211-c.387)
	[M13-Rev]-tcccatctgaaaaacaaggtag		
D	[-21M13]-ttgctacctggcgttttctaa	4	(c.388-c.472)
	[M13-Rev]-cttgccgcagaaaagagaaa		
E	[-21M13]-catgcagattggagaacgag	5 & 6	(c.473-c.687)
	[M13-Rev]-atggctgcccacatcatt		
F	[-21M13]-ggaaggcaagtggagctatg	7 & 8	(c.688-c.894)
	[M13-Rev]-tgggcacatgtagacctgaa		
G	[-21M13]-tttcttcggcatgcaataca	9	(c.895-c.1043)
	[M13-Rev]-ctgccaagaggtcaagaaaga		
H	[-21M13]-gcagagtcctgaagaatgagc	10 & 11	(c.1044-c.1278)
	[M13-Rev]-ccccaagagacaagtaagcag		
	Internal rev seq primer cagcaacccatctcacat		
I	[-21M13]-gggcagagttgattgcttgt	12 & 13	(c.1279-c.1406)
	[M13-Rev]-gccagccacttagtttggat		
J	[-21M13]-ttgtggaatgcaagattctg	14 & 15	(c.1407-c.1655)
	[M13-Rev]-tgatttgtgcaccttctgga		
	Internal rev seq primer cagaaacaaagtagtcttc		
K	[-21M13]-gagtgcctgtgtctttttgg	16 & 17	(c.1656-c.1875)
	[M13-Rev]-gaacctgggagatggaggtt		
	Internal forw seq primer ctgagtggatccatggcattg		
L	[-21M13]-tcaaatggtacatgctttgaaga	18	(c.1876-c.2016)
	[M13-Rev]-gggtttgggatcagggagta		
M	[-21M13]-ttctgttgctttctttgttcg	19	(c.2017-c.2184)
	[M13-Rev]-caatcaatcataagattcctgaaaaa		
N	[-21M13]-gacaggaaaacctgctaggc	20	(c.2185-c.2280)
	[M13-Rev]-gactcccatcactgggtcac		
O	[-21M13]-ttgaggtgtatttgaagttcagtg	21 & 22	(c.2281-c.2485)
	[M13-Rev]-acatcttcccattcctgctg		
	Internal rev seq primer ctacccacattagagcaacg		
P	[-21M13]-ggcaggaggagatgacagac	23	(c.2486-c.2662)
	[M13-Rev]-actcagccaccttggaactg		
Q	[-21M13]-aaaatgcctaggcctccaag	24	(c.2663-c.*187)
	[M13-Rev]-gtggcttgcatctcctgttc		
R	5^′^-tctccaagggtcttcaccac-3^′^		c.47-c.66
	5^′^-agaatgccaccagctttgac-3^′^		c.236-c.217
S	5^′^-agttcctcaggcagagtcca-3^′^		c.2421-c.2440
	5^′^-ggctgaaaagccattgatgt-3^′^		c.2607-c.2588
T	5^′^-gccttccatcccagtttctt-3^′^		c.–116-c.–97
	5^′^-ctgcatctctcaccacagga-3^′^		c.1323-c.1342
	Internal seq. primer 5^′^-tgcatttttctcccacacaa-3^′^		c.318-c.337
	Internal seq. primer 5^′^-cccaaactggagacctcaga-3^′^		c.755-c.774
U	5^′^-gaatgctttggagacatgctt-3^′^		c.1237-c.1257
	5^′^-ggtgtattgcctgggaagaa-3^′^		c.*23-c.*42
	Internal seq. primer 5^′^-ctgcaagctacatcacaccag-3^′^		c.1724-c.1744
	Internal seq. primer 5^′^-tggcatttcttctgctcaca-3^′^		c.2136-c.2155

^1^All forward and reverse primers were tagged with a –21M13 (5^′^-TGTAAAACGACGGCCAGT-3^′^) sequence or M13rev (5^′^-CAGGAAACAGCTATGACC-3^′^) sequence.

RNA was isolated from fibroblast pellets using Trizol extraction. cDNA was synthesized by using the Superscript II Reverse Transcriptase Kit (Invitrogen, Carlsbad, CA, USA). Quantitative real-time PCR analysis of AASS expression in fibroblasts was performed using the LC480 Sybr Green I Master mix (Roche) and primer sets R and S to amplify the 5^′^ and 3^′^ part of the AASS cDNA, respectively. In patient 1, 2 and 5 the complete AASS cDNA was amplified and sequenced using primer sets T and U (Table 2).

### Comparative genomic hybridization

Comparative genomic hybridization (CGH) was performed using an Agilent 180K oligo-array (Amadid 023363, Agilent technologies Inc, Santa Clara, CA, USA) as described before [19]. All genome coordinates mentioned are according to human genome build 19.

### Immunoblot analysis

Fibroblast homogenates were prepared in PBS using sonication (twice on ice, 40J at 8W output). Samples were further prepared according to the instructions for electrophoresis of NuPAGE Bis-Tris mini gels (4–12%; Life Technologies) using sample buffer for denaturation and reduction of the protein disulfide bonds. Equal amounts of protein (30 μg) were loaded. A rabbit anti-AASS antibody (1 in 500 dilution) raised against amino acids 528 to 649 of the protein was obtained from Sigma-Aldrich (HPA020728). Antibodies were visualized using IRDye 800CW or IRDye 680RD anti-rabbit secondary antibodies and the Odyssey Infrared Imaging System (Li-Cor Biosciences).

## Results and discussion

Hyperlysinemia was diagnosed based on elevated plasma lysine levels in 8 patients with distinct neurological features whose material was sent to a laboratory for metabolic screening and confirmatory testing (Table 1). All patients had no detectable LKR and SDH activity in fibroblasts. For molecular genetic analysis, we sequenced all 24 exons including the flanking exon/intron boundaries of *AASS* by using standard Sanger sequencing techniques. To determine whether the identified mutations affect the AASS protein levels, we performed immunoblot analysis in fibroblast homogenates (Figure 1B).

Cases 1 and 2 are compound heterozygous for a transition c.194G>A in exon 2, and a transversion c.1256T>G in exon 11. Both substitutions change conserved amino acids, arginine at position 65 into glutamine (p.R65Q) and leucine at position 419 into arginine (p.L419R). In case 3 the c.194G>A mutation was homozygous, consistent with the reported consanguinity of the parents. Immunoblot analysis revealed that AASS protein was absent in case 1 and 3, indicating that both mutations primarily affect protein levels (Figure 1B). In case 4 no mutations were found in exons 2 to 24, but we failed to amplify exon 1 indicating the presence of a deletion. Indeed, no AASS cDNA and protein could be detected in this case (Figure 1B&C), indicating that this deletion interferes with expression of AASS. In case 5 we found two mutations, a heterozygous transition c.460G>A in exon 4 which changes a conserved alanine in the LKR domain into threonine (p.A154T), and a duplication of 1 basepair c.2076dup in exon 19, which creates a frame shift at amino acid position 693 resulting in a stop codon after 9 triplets (p.P693SfsX10). The c.460G>A mutation appeared homozygous at the cDNA level, indicating nonsense-mediated decay of the c.2076dup mRNA (Figure 1D). Indeed no immunoreactive protein with the predicted Mw of the truncated protein (78kDa) was observed upon immunoblotting. The detected AASS with a Mw of 102kDa is therefore the p.A154T mutant protein. In case 6, we found a homozygous transition c.2155A>G in exon 19, which changes threonine at position 719 into alanine (p.T719A). This mutation affects a highly conserved amino acid in the SDH domain of AASS. The activity of LKR is most likely at least partially conserved, which is evidenced by the fact that this patient displayed hyperlysinemia and saccharopinuria. Unfortunately, the available lymphoblasts of this patient were not suited for follow up analysis, because AASS activity and protein are undetectable in this cell type.

In cases 7 and 8 no mutations were found in exons 1 to 19, but we failed to amplify exon 20–24 indicating the presence of a large deletion. Despite the fact that the 5^′^ part of the AASS cDNA could be detected (Figure 1C), no AASS protein was present in both cases (Figure 1B). In order to determine the size of this deletion, we performed CGH in case 7. This analysis revealed a homozygous deletion of 126kb at 7q31.32 (karyotype arr7q31.32(121590497–121716631)x0) involving two genes, *AASS* and *PTPRZ1*. Although not exactly known, the deletion breakpoint for *AASS* must be located in intron 19 or exon 20. *PTPRZ1* or protein tyrosine phosphatase, receptor-type, Z polypeptide 1 is encoded by 30 exons and spans 193 kbp. The breakpoint for *PTPRZ1* lies in exon 2 or intron 2, which will prevent the expression of functional PTPRZ1. PTPRZ1 is a protein tyrosine phosphatase, which is mainly expressed in the developing and mature brain (astrocytes, oligodendrocyte precursors, immature and mature oligodendrocytes). Binding with a variety of cell adhesion and matrix molecules affect intracellular phosphatase activity [20,21]. Although PTPRZ1 knockout mice have no gross anatomical alterations in their nervous system or other organs, they do display disturbed motor coordination and nociception and have decreased recovery from demyelinating lesions [22,23]. Thus the physiological role of PTPRZ1 may be in oligodendrogenesis, promotion of neurite outgrowth and glial adhesion [21]. Our data indicate that cases 7 and 8 have a novel contiguous gene deletion syndrome that was biochemically diagnosed as hyperlysinemia. We speculate that the severe neurological disease in these cases is caused by a loss of PTPRZ1 function.

In addition to the single mutation reported before, we now describe 7 novel mutations, one of which was present in two unrelated patients (p.R65Q). All missense mutations affect conserved amino acids and are not present in the SNP or 1000 genomes database. The causal nature of these mutations is further evidenced by deficient LKR and SDH activities in all patients’ fibroblasts and the undetectable AASS protein levels in all but one case. In our cohort of patients, hyperlysinemia tracks with distinct neurological features. This could reflect the selection bias in our cohort, since amino acid analyses are mainly performed in patients with neurological abnormalities. Although, the neurological phenotype segregated with the biochemical abnormalities in all studied siblings, this is only weak evidence for causality in recessive disorders with single cases and consanguinity. In addition, there is a broad range of clinical phenotypes in the group of patients in which only AASS is defective. Patient 1 and 2 are more severely affected than patient 3 and 4, despite the fact that all cases appear to have no functional AASS protein. We suggest that the pathophysiology of AASS deficiency may be better addressed in a model organism such as an *Aass* knockout mouse. Population-based studies in which the prevalence of hyperlysinemia is measured (through metabolomics) in conjunction with genotyping and clinical phenotyping might provide additional insight.

## Conclusions

We conclude that in our cohort of 8 cases, hyperlysinemia is caused by mutations in the *AASS* gene. In two patients originating from one family, the hyperlysinemia was caused by a contiguous gene deletion syndrome affecting *AASS* and *PTPRZ1*. Our findings illustrate the importance of detailed biochemical and genetic studies in any hyperlysinemia patient.

## Abbreviations

LKR: Lysine-ketoglutarate reductase; SDH: Saccharopine dehydrogenase; AASS: α-aminoadipic semialdehyde synthase; MRI: Magnetic resonance imaging; CGH: Comparative genomic hybridization; PTPRZ1: Protein tyrosine phosphatase, receptor-type, Z polypeptide 1.

## Competing interests

The authors declare that they have no competing interests.

## Authors’ contributions

SH, RW and MD conceived and designed the study. SH drafted the manuscript. HtB carried out the molecular genetic studies. SD and JR carried out the biochemical studies. AK performed the CGH. JK, PA, MH, MB, TC, JZ, JOS, BT, RJ and MD were involved in the clinical evaluation, diagnosis and follow-up of the patients. All authors read and approved the final manuscript.

## References

[B1] Markovitz PJ Chuang DT Cox RP Familial hyperlysinemias. Purification and characterization of the bifunctional aminoadipic semialdehyde synthase with lysine-ketoglutarate reductase and saccharopine dehydrogenase activities J Biol Chem 1984 259 11643 11646 6434529

[B2] Sacksteder KA Biery BJ Morrell JC Goodman BK Geisbrecht BV Cox RP Gould SJ Geraghty MT Identification of the alpha-aminoadipic semialdehyde synthase gene, which is defective in familial hyperlysinemia Am J Hum Genet 2000 66 1736 1743 10775527 10.1086/302919 PMC1378037

[B3] Mills PB Struys E Jakobs C Plecko B Baxter P Baumgartner M Willemsen MA Omran H Tacke U Uhlenberg B Mutations in antiquitin in individuals with pyridoxine-dependent seizures Nat Med 2006 12 307 309 16491085 10.1038/nm1366

[B4] Goodman SI Kratz LE DiGiulio KA Biery BJ Goodman KE Isaya G Frerman FE Cloning of glutaryl-CoA dehydrogenase cDNA, and expression of wild type and mutant enzymes in Escherichia coli Hum Mol Genet 1995 4 1493 1498 8541831 10.1093/hmg/4.9.1493

[B5] Danhauser K Sauer SW Haack TB Wieland T Staufner C Graf E Zschocke J Strom TM Traub T Okun JG DHTKD1 Mutations Cause 2-Aminoadipic and 2-Oxoadipic Aciduria Am J Hum Genet 2012 91 1082 1087 23141293 10.1016/j.ajhg.2012.10.006 PMC3516599

[B6] Duran M Blau N, Duran M, Gibson KM Amino acids Laboratory Guide to the Methods in Biochemical Genetics 2008 Berlin-Heidelberg: Springer-Verlag 53 89

[B7] Hoffmann GF Kolker S Saudubray JM, van den Berghe G, Walter JH Cerebral organic acid disorders and other disorders of lysine catabolism Inborn Metabolic Diseases 2012 5 Berlin-Heidelberg: Springer-Verlag 333 346

[B8] Saudubray JM Rabier D Biomarkers identified in inborn errors for lysine, arginine, and ornithine J Nutr 2007 137 1669S 1672S 17513445 10.1093/jn/137.6.1669S

[B9] Dancis J Hutzler J Cox RP Woody NC Familial hyperlysinemia with lysine-ketoglutarate reductase insufficiency J Clin Invest 1969 48 1447 1452 5796356 10.1172/JCI106110 PMC322371

[B10] Kamoun P Richard V Rabier D Saudubray JM Plasma lysine concentration and availability of 2-ketoglutarate in liver mitochondria J Inherit Metab Dis 2002 25 1 6 11999975 10.1023/a:1015195009330

[B11] van der Heiden C Brink M de Bree PK van Sprang FJ Wadman SK de Pater JM van Biervliet JP Familial hyperlysinaemia due to L-lysine alpha-ketoglutarate reductase deficiency: results of attempted treatment J Inherit Metab Dis 1978 1 89 94 116084 10.1007/BF01805679

[B12] Woody NC Pupene MB Excretion of pipecolic acid by infants and by patients with hyperlysinemia Pediatr Res 1970 4 89 95 5417004 10.1203/00006450-197001000-00011

[B13] Cederbaum SD Shaw KN Dancis J Hutzler J Blaskovics JC Hyperlysinemia with saccharopinuria due to combined lysine-ketoglutarate reductase and saccharopine dehydrogenase deficiencies presenting as cystinuria J Pediatr 1979 95 234 238 571908 10.1016/s0022-3476(79)80657-5

[B14] Dancis J Hutzler J Ampola MG Shih VE van Gelderen HH Kirby LT Woody NC The prognosis of hyperlysinemia: an interim report Am J Hum Genet 1983 35 438 442 6407303 PMC1685659

[B15] Gregory JW Beail N Boyle NA Dobrowski C Jackson P Dietary treatment of hyperlysinaemia Arch Dis Child 1989 64 716 720 2499273 10.1136/adc.64.5.716 PMC1792052

[B16] Seminotti B Leipnitz G Amaral AU Fernandes CG de Bortoli da Silva L Tonin AM Vargas CR Wajner M Lysine induces lipid and protein damage and decreases reduced glutathione concentrations in brain of young rats Int J Dev Neurosci 2008 26 693 698 18691648 10.1016/j.ijdevneu.2008.07.011

[B17] Tonin AM Ferreira GC Schuck PF Viegas CM Zanatta A Leipnitz G Seminotti B Duvall Wannmacher CM Wajner M Inhibition of creatine kinase activity by lysine in rat cerebral cortex Metab Brain Dis 2009 24 349 360 19370404 10.1007/s11011-009-9131-z

[B18] Seminotti B Fernandes CG Leipnitz G Amaral AU Zanatta A Wajner M Neurochemical evidence that lysine inhibits synaptic Na+, K+-ATPase activity and provokes oxidative damage in striatum of young rats in vivo Neurochem Res 2011 36 205 214 20976553 10.1007/s11064-010-0302-4

[B19] Barge-Schaapveld DQ Maas SM Polstra A Knegt LC Hennekam RC The atypical 16p11.2 deletion: a not so atypical microdeletion syndrome? Am J Med Genet A 2011 155A 1066 1072 21465664 10.1002/ajmg.a.33991

[B20] Lamprianou S Chatzopoulou E Thomas JL Bouyain S Harroch S A complex between contactin-1 and the protein tyrosine phosphatase PTPRZ controls the development of oligodendrocyte precursor cells Proc Natl Acad Sci USA 2011 108 17498 17503 21969550 10.1073/pnas.1108774108 PMC3198311

[B21] Mohebiany AN Nikolaienko RM Bouyain S Harroch S Receptor-type tyrosine phosphatase ligands: looking for the needle in the haystack FEBS J 2013 280 388 400 22682003 10.1111/j.1742-4658.2012.08653.x PMC3753797

[B22] Harroch S Furtado GC Brueck W Rosenbluth J Lafaille J Chao M Buxbaum JD Schlessinger J A critical role for the protein tyrosine phosphatase receptor type Z in functional recovery from demyelinating lesions Nat Genet 2002 32 411 414 12355066 10.1038/ng1004

[B23] Lafont D Adage T Greco B Zaratin P A novel role for receptor like protein tyrosine phosphatase zeta in modulation of sensorimotor responses to noxious stimuli: evidences from knockout mice studies Behav Brain Res 2009 201 29 40 19428613 10.1016/j.bbr.2009.01.025

